# Routine Detection of Viruses Through Metagenomics: Where Do We Stand?

**DOI:** 10.4269/ajtmh.24-0652

**Published:** 2024-12-24

**Authors:** Svetoslav Nanev Slavov

**Affiliations:** Center for Viral Surveillance and Serological Evaluation (CeVIVAs), Butantan Institute, São Paulo, Brazil

Significant advances in next-generation sequencing have established viral metagenomics as a powerful tool for detecting unsuspected or unknown viruses, particularly in clinical settings where conventional diagnostic methods have failed. This unbiased approach, which does not require prior knowledge of the etiological agent, has advantages over routine viral diagnostics by eliminating the need for specific primers, probes, specialized culture conditions, or media. It provides a comprehensive view of all genomic sequences present in a given sample, thus overcoming the limitations of traditional molecular tests, which are restricted to specific pathogens or rely on diagnostic panels for a number of agents. In the study by Kone et al. in this issue of the *American Journal of Tropical Medicine and Hygiene*, virome capture metagenomic sequencing (using the VirCapSeq-VERT assay with multiple virus concentration probes) was applied in a conventional laboratory in Bamako, Mali, to enhance the understanding of causes of acute febrile illnesses in the country.^1^^,^^2^ Clinically important viruses such as measles virus, SARS-CoV-2, hepatitis B virus, parvovirus B19, adenovirus, and human herpesvirus were identified, alongside viruses that are part of the normal blood virome (e.g., anelloviruses). These findings, beyond their diagnostic potential, provide insight into the spread of these viral agents in Mali. The study did not identify emerging viruses that might cause hemorrhagic fevers or other serious clinical conditions.

One of the most significant applications of metagenomics is the elucidation of etiologies of acute illnesses. No prior knowledge of the infecting pathogen is required. For example, in a case of severe pulmonary infection, where the patient did not respond to conventional antibiotic therapy, the identification of *Leptospira interrogans* through metagenomics enabled targeted treatment, resulting in a favorable patient outcome.^3^ Focusing on viruses, viral metagenomics has revolutionized the field of viral genotyping and phylogenetic analysis by providing critical genomic information, offering insights into viral evolution and the acquisition of mutations associated with increased virulence. This capability is especially valuable for public health authorities, enabling rapid responses to emerging pathogens, particularly during outbreaks and pandemics, by facilitating the swift identification of new viral strains. In this context, next-generation sequencing technologies have proven particularly useful in characterizing viral outbreaks, such as the Zika virus outbreak in Brazil^4^ and the emergence of virulent strains during recent Ebola outbreaks in Africa.^5^^,^^6^

A major challenge of metagenomic analysis, particularly in resource-limited countries, is the high cost of reagents and bioinformatics analysis, making this method more expensive compared to traditional diagnostic tools. Additionally, the required wet lab and computational infrastructure is costly and requires highly skilled personnel, which is often lacking in these settings. As a result, metagenomic studies are typically conducted in collaboration with institutions that possess the necessary infrastructure, leading to dependency on these collaborations. This reliance can be problematic for routine diagnostics, potentially causing significant delays in obtaining results. Careful consideration must also be given to the reagents used for sample preparation, as they are continually evolving and must be chosen based on the sample type. For routine diagnosis, operational procedures must adhere to strict requirements and be approved by local regulatory agencies.^7^ The time required for sample processing is also a significant limitation, as it involves multiple steps, including sample extraction, viral nucleic acid concentration, amplification, library preparation, next-generation sequencing, and bioinformatic analysis. From the acquisition of the clinical sample to the final bioinformatic analysis, the entire process can take weeks or months, making it unsuitable for routine diagnosis, particularly in acute and severe illnesses. Assay sensitivity (limit of viral detection) is another critical factor that must be carefully evaluated when applying metagenomics, as there are no standardized protocols for its optimization. As a result, infections with low viral loads might be missed. Furthermore, it is important to recognize that the identification of a pathogen genome does not necessarily indicate an active infection. Additionally, a major component (sometimes exceeding 90% of the total virome abundance) of viral metagenomic sequences consists of commensal viruses, such as anelloviruses or other circular viruses, which are probably of no clinical importance.^8^

In addition to technical difficulties of metagenomic assays, bioinformatic analysis poses significant challenges. First, it requires highly specialized computational infrastructure, bioinformatics experts, and technical support for optimal functioning of the computational workflow, all of which are often not readily available in resource-limited countries. Second, there is variability in analytical pipelines between laboratories, and the use of different software for viral identification may lead to varying levels of sensitivity, in particular depending on the virus databases utilized. This can result in differences in measures of viral abundance, even when testing the same sample. Third, the interpretation of results, which may include the presence of artifacts and contaminants, can be difficult.^9^ These may arise from various sources, including laboratory surfaces. Consequently, additional pipelines may be required to exclude such sequences, or molecular confirmation of identified viruses of interest may be mandatory.^10^ Currently, a significant proportion of sequences obtained by metagenomic analysis remain unclassified, falling into what is known as "dark matter", highlighting that classification is limited to known viruses, while identification of emerging viruses is difficult.^11^

While viral metagenomics has revolutionized our understanding of emerging viruses and facilitated their discovery, this methodology still presents significant technical and analytical challenges for routine diagnostic applications, particularly in resource-limited countries. These challenges include, but are not limited to, high cost, complex bioinformatics requirements, and difficulties in linking classified viral abundance to clinically relevant pathogens. Overcoming these limitations in the future will be essential for integrating metagenomics as a routine diagnostic technique, which should enhance our ability to respond to acute illnesses and outbreaks of unknown origin and to improve public health surveillance.
